# PDBMD2CD: providing predicted protein circular dichroism spectra from multiple molecular dynamics-generated protein structures

**DOI:** 10.1093/nar/gkaa296

**Published:** 2020-04-28

**Authors:** Elliot D Drew, Robert W Janes

**Affiliations:** School of Biological and Chemical Sciences, Queen Mary University of London, Mile End Road, London, E1 4NS, UK; School of Biological and Chemical Sciences, Queen Mary University of London, Mile End Road, London, E1 4NS, UK

## Abstract

PDBMD2CD is a new web server capable of predicting circular dichroism (CD) spectra for multiple protein structures derived from molecular dynamics (MD) simulations, enabling predictions from thousands of protein atomic coordinate files (e.g. MD trajectories) and generating spectra for each of these structures provided by the user. Using MD enables exploration of systems that cannot be monitored by direct experimentation. Validation of MD-derived data from these types of trajectories can be difficult via conventional structure-determining techniques such as crystallography or nuclear magnetic resonance spectroscopy. CD is an experimental technique that can provide protein structure information from such conditions. The website utilizes a much faster (minimum ∼1000×) and more accurate approach for calculating CD spectra than its predecessor, PDB2CD (1). As well as improving on the speed and accuracy of current methods, new analysis tools are provided to cluster predictions or compare them against experimental CD spectra. By identifying a subset of the closest predicted CD spectra derived from PDBMD2CD to an experimental spectrum, the associated cluster of structures could be representative of those found under the conditions in which the MD studies were undertaken, thereby offering an analytical insight into the results. PDBMD2CD is freely available at: https://pdbmd2cd.cryst.bbk.ac.uk.

## INTRODUCTION

Circular Dichroism (CD) spectroscopy is a widely used technique to explore and examine aspects of protein structure in solution. CD data can be employed to determine the secondary structure content of a protein and hence, whether a protein is correctly folded or not. Notably, it is also a dynamic technique able to monitor any resultant structural changes that arise when ambient solution conditions are altered, for example by temperature or pH. Additionally, identifying changes in structure following the binding of ligands, cofactors or other proteins is also possible. So the technique offers a highly versatile approach to monitoring the dynamics of structural changes of proteins in solution when it is not possible via other techniques such as crystallography, or even solution nuclear magnetic resonance (NMR) studies, to make such comparisons.

Molecular dynamics (MD) simulation studies, specifically those on proteins, can be used to examine the structure and dynamics of complex macromolecular systems as they evolve over time. As the name suggests, these are *in silico* simulations capable of offering atomistic and dynamic insight on systems, even where there are no experimental techniques available to obtain such data directly. In these studies, it is important to validate the MD results whenever possible, by examining the extent of the match between data generated computationally and any comparable data obtained from a supporting experimental technique. Data that match well lends weight, and hence validity, to other properties obtained from an MD study where those specific results cannot be supported by direct experimental means. The use of MD for studying interactions involving proteins is growing year on year, so it is all the more important to have an experimental technique which is versatile enough to provide the necessary supporting data to match with the MD results. CD spectroscopy is readily available as such a technique.

Previously we developed a web server, PDB2CD (1), dedicated to producing predictions of CD spectra from protein atomic coordinates. That package has fulfilled an important role in providing users with meaningful predicted CD spectra of proteins where obtaining an experimental CD spectrum was not possible, often enabling comparison of this against an actual spectrum from a related protein (2–6).

However, PDB2CD was not envisioned to cater for multiple input files due to the inherent ‘rate determining step’, that of performing the structure comparisons between the query structure and those in the reference set of proteins used, the SP175 (7) ‘gold standard’ set available in the Protein Circular Dichroism Data Bank (PCDDB) (8). This meant that to generate predicted CD spectra using PDB2CD, structures derived in an NMR ensemble had to be input individually. For structures generated from MD studies, only the average, or most representative structure from the trajectories would be entered (5,6). To provide a site with additional analysis tools capable of dealing with generating predicted CD spectra from multiple protein structure input files in an efficient and accurate way, the PDBMD2CD web server has been created.

## THE PDBMD2CD SERVER

The PDBMD2CD web server is a user-friendly resource designed with the aim of making it available to a wider user community. Specifically, the focus has been to facilitate the input of multiple protein structure entries from which predicted CD spectra are generated for each one. Our aims were to optimize on speed of delivering the results whilst retaining, and improving, the accuracy levels of CD spectra prediction. Enabling multiple structure input means that all selected structures sampled from MD trajectories as a representative set can be used to generate information over the entire time-course of the study, rather than using only one ‘representative’ structure (5,6).

An overview of the methodology behind the PDBMD2CD package is presented here, with more details in the Supplementary Data. PDBMD2CD employs a combination of two different approaches: The first uses basis spectra that represent seven secondary structural types derived from a least squares regression of the CD signal (Supplementary Figure S1 and Supplementary Table S2) from structures in our 83 protein reference set (a set derived from the high-quality SMP180 (9) which has a broader range of proteins incorporating both soluble and membrane proteins, and shown in Supplementary Table S1). The second creates a basis set of spectra from structures in the reference set with the closest secondary structure content to the query protein, estimating each basis spectra's weighting in the final prediction through a multivariate optimization of the contributing spectra's summed secondary structure content. The two predicted spectra from these approaches are then averaged to give the final prediction, resulting in better accuracy than either method alone.

**Figure 1.**
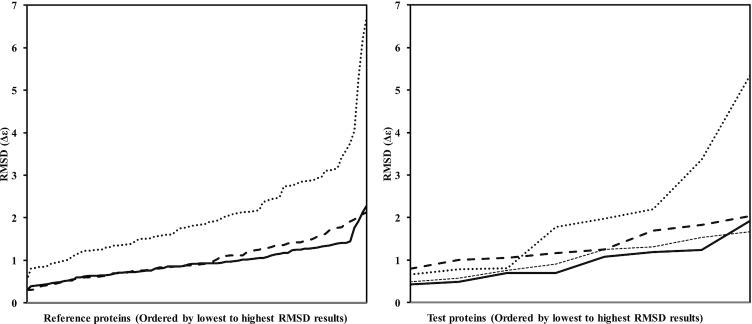
Plots of the RMSD values between calculated spectra for DichroCalc (dotted line), PDB2CD (thick dashed line), SESCA (right hand panel thin dashed line) and PDBMD2CD (solid line) for the Reference proteins in the leave-one-out cross-validation, (left hand panel) and for the test proteins (right hand panel).

**Figure 2.**
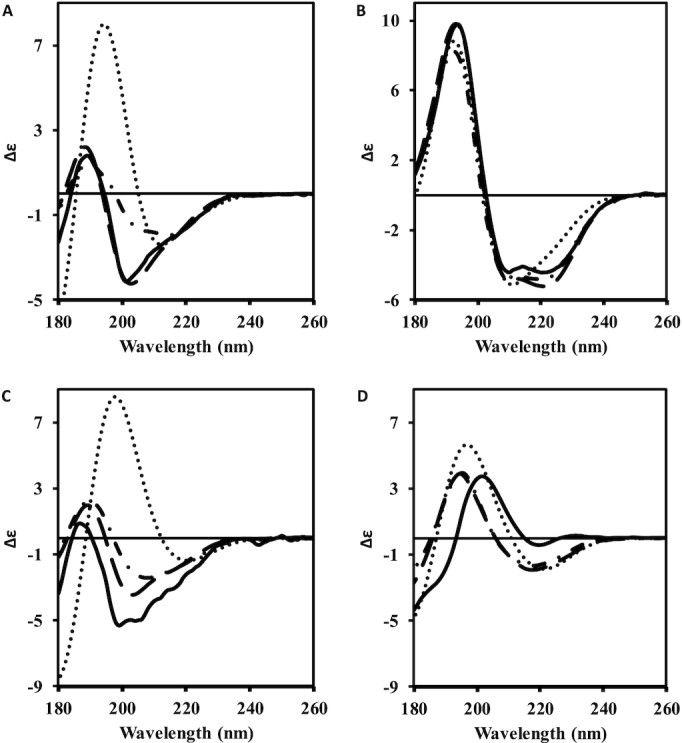
Examples of predicted spectra from DichroCalc (dotted line), PDB2CD (dot-dash line) and PDBMD2CD (dashed line) plotted against the experimentally determined spectrum (18) (solid line) obtained from the PCDDB (8). Two good PDBMD2CD examples are: (**A**) human dUTPase (PDB code: 1Q5U) and (**B**) 3-isopropylmalate dehydrogenase (PDB code: 2Y3Z). Two poorer examples are: (**C**) Ecotin (PDB code: 1ECZ) and (**D**) Beta-2-microglobulin (PDB code: 2YXF).

Testing of PDBMD2CD was performed using leave-one-out (LOO) cross-validation on the training set using the root mean square deviation (RMSD) between predicted and experimental spectra as a performance metric. In addition, predictions were made on a separate test set of eight structures derived from the PCDDB (Table 1 and Supplementary Table S3). Summarized data comparisons are presented for PDB2CD (1), the DichroCalc web server (10) and SESCA (for the test set only) (11), a downloadable, command-line python package. Results from PDBMD2CD (Table 1) were in good agreement overall with the experimental spectra and showed significant improvements over existing *ab initio* and empirical methods, including PDB2CD. Figure 1 shows comparisons of the RMSD differences between the experimental and predicted CD spectra for PDBMD2CD, PDB2CD and DichroCalc, ordered from the lowest to highest RMSD values both for the LOO cross-validation results and for the test set of proteins. Examples of predicted CD spectra for the test set structures are presented in Figure 2. Two of the better-predicted spectra are shown in Figure 2A and B, whilst two of the poorer predictions are shown in Figure 2C and D.

**Table 1.** Left panel, cross-validation leave-one-out (LOO) data for the 83 proteins in the reference set and comparison of predicted and right panel, experimental CD spectra of eight test proteins

LOO reference set	RMSD	Test set (eight spectra)	RMSD
**PDBMD2CD**	**0.940 (0.394)**	**PDBMD2CD**	**0.962 (0.496)**
PDB2CD (SMP180)	1.026 (0.468)	PDB2CD (SMP180)	1.347 (0.443)
DICHROCALC	2.062 (1.111)	DICHROCALC	2.111 (1.596)
		SESCA – (DSSP-1 basis set)	1.055 (0.442)

The average RMSD values for PDBMD2CD, PDB2CD and DichroCalc for the LOO data, and included is SESCA for the test set of proteins (lowest values are the best and highlighted in bold). Standard deviations are given in brackets.

## INPUT AND OUTPUT

### Input

PDBMD2CD takes as input Protein Data Bank (PDB) structure files, either in PDB or mmCIF format. Single or multiple structure files may be uploaded. To minimize wait time caused by upload speeds, large numbers of files can be uploaded as a single compressed (zip, bzip or tar.gz archive) file. Alternatively, it is possible to use a PDB code, or multiple codes separated by commas, placed into the box provided, so the structure files corresponding to those codes are fetched from the RCSB PDB (12) servers. Although the main focus has been on files from MD simulations, multiple files from other sources can also be used. For example, these could be from an NMR ensemble of structures where it would be of interest to see the range of conformations obtained, particularly where flexibility in the structure might lead to differing clusters of structures; CD could be used to arbitrate between these possible outcomes. Another illustration of potential usage is in homology studies, taking the structures of a range of related proteins from the PDB and then comparing their predicted spectra to that of an experimental one to ascertain which of the homologous structures most resembles that of the experimental protein.

### Output

**Figure 3.**
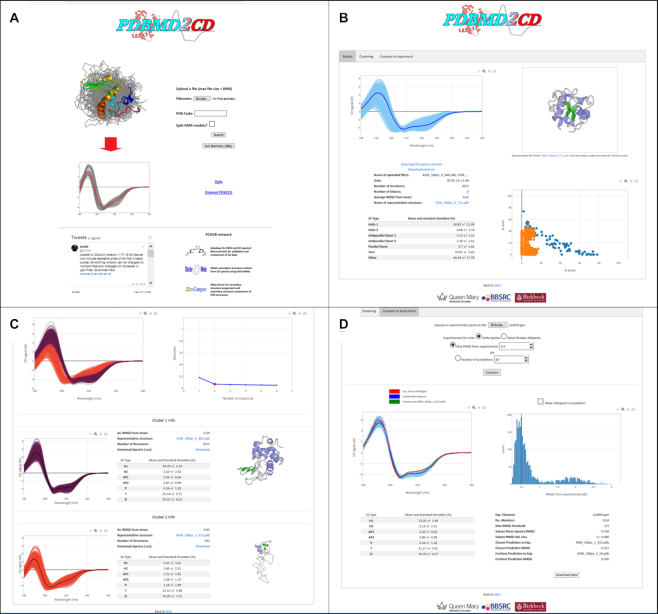
Example pages from the PDBMD2CD web server. Panel (**A**) is the landing input page; panel (**B**) the ‘Results’ page showing all the predicted spectra (in light blue in the left pane together with the most representative spectrum of the set in dark blue), the most representative structure of the set (in the right upper pane), the secondary structure alpha helix versus beta sheet content in the right hand lower pane, the blue being for the reference set, the orange for the input structures (2672 here) and associated secondary structure information (in the lower left pane); panel (**C**) is the ‘Clustering’ page illustrating the results from a k-mean = 2 clustering (shown in the right hand pane), where, below this, the spectra have been clustered into the purple first cluster set, and the dark orange second cluster set, and there are associated secondary structure information and the most representative structures associated with each cluster also shown in the panes to the right of the plots; Panel (**D**) is the ‘Compare to Experiment’ page and shows the information obtainable from comparison to an input experimental spectrum. Here a subset of spectra is being shown with an RMSD smaller or equal to 0.5 as maximum away from the experimental spectrum. The spectra are shown in the left pane in light blue, with the experimental spectrum in red, the subset mean in dark blue and the closest spectrum to the experimental in green. A histogram plot showing the RMSD distribution of predicted spectra distanced from the experimental spectrum is shown in the right hand pane with a red line indicating the RMSD/number of chosen spectra in the current subset. Below this are secondary structure and associated data pertinent to the current chosen subset.

PDBMD2CD creates three output pages which may be accessed through a tabbed menu. These are the ‘Results’, which is the default page, ‘Clustering’ and ‘Compare to Experiment’ pages. Each page has specific types of analyses for the multiple predicted spectra. An example of how these pages appear, together with the home webpage, is given in Figure 3 and is discussed in the ‘Case Study’ section.

### Results tab

On this page are all the generated CD spectra together with the mean of these spectra in one interactive graphic. To the right of that is an interactive graphics pane showing the structure most representative of the set of structures entered. Below this is a secondary structure plot of alpha helix versus beta sheet content as derived by the ‘Dictionary of Secondary Structure of Proteins’ (DSSP) definition (13), showing in blue the data for the reference set of structures used and in orange the positions of each of the structures uploaded for generating their CD spectra. To the left of this are data associated with the generated CD spectra: the names of the structures used in the predictions, the date and time of the run, the number of structures uploaded, a report on the number of any failures within this set (with a pull down box below this value indicating which these are if there are any), the average RMSD of the generated spectra from the mean of the set and the name of the most representative structure file (the structure shown in the upper right pane) in PDB format. There are links available to this file in this table and also below the interactive structure pane. The spectra are downloadable both as .zip and .csv files. Below this are the mean values for the secondary structure content present in the structures, with an associated standard deviation. These secondary structure features are: Helix 1, Helix 2, Antiparallel Sheet 1, Antiparallel Sheet 2, Parallel Sheet, Turn and Other (defined as the content of what is left that is not defined by those terms above it) such that the total adds to 100% (defined in the Supplementary Data).

### Clustering tab

On this page it is possible to group the predicted spectra into clusters using the k-means clustering (14) method (see Supplementary Data for the full explanation of this approach). Once again, the overall set of predicted spectra are presented on the top left of the page. On the right of this is an ‘elbow’ plot, which allows visual estimation of the optimal number of clusters within this set of spectra by identifying the smallest number of clusters which account for the largest variation in the data. The elbow plot shows the distortion, calculated as the mean Euclidean distance between the cluster members and the centroids generated for values of k from one through to six inclusive. The largest deviation from linearity between three contiguous k-mean cluster values will produce a bend forming the ‘elbow’ and the middle value of the three represents the most likely number of clusters present in the data set (see also the Supplementary Data). The plot gives a guide to the number of clusters present in the data, but the user needs to make the ultimate judgement on what might be considered the best number of clusters.

As the k value is modified by the user (from a pull down box at the top of the ‘Clustering’ page), so the spectra within the main representation are coloured according to the numbers of clusters identified, and below this main plot are the individual clusters similarly coloured. Each cluster has separate data associated with them which includes to the right of the plot the average RMSD of the clustered spectra from its mean, the identity (and a link to it) of the representative structure of that cluster, the number of structures within that cluster, the average secondary structure content and a download link to the spectra associated within the cluster, in .csv format. To the right of this is an interactive pane displaying the most representative structure of that cluster.

### Compare to Experiment tab

This page enables comparisons to be made between the predicted spectra and a user-provided experimentally-derived spectrum. The user file can be uploaded in many standard text-based output formats (produced by different CD instruments), as an output file from the Synchrotron Radiation CD (SRCD) beamlines and as a generic two-column format (wavelength versus CD data). Uploaded spectra can be in either Delta Epsilon units (as default) or in Mean Residue Ellipticity (chosen by selecting a radio button), which in this case the package converts into Delta Epsilon.

The RMSDs between the experimental and each of the predicted CD spectra are used as the measure for comparison. The user may choose between comparing using an RMSD threshold or by specifying the number of predicted spectra to show that are closest to the experimental spectrum. By default, this RMSD value (which may be modified by the user), is initially set at 0.5, or to half the maximum RMSD value if that value is smaller than 0.5. As the user choses a maximum RMSD value so the value showing of the number of closest predicted spectra is updated to that which equates to the chosen RMSD value, and the reciprocal of this happens if a number of closest spectra is chosen instead. Each comparison will generate a subset of the predicted spectra as a result. To the left below this section is a plot displaying the currently chosen subset of spectra together with the experimental spectrum in red, the mean prediction of the subset in blue, and the closest predicted spectrum to the experimental spectrum in green. To the right of this is a pane showing a histogram plot of the distribution of predicted spectra RMSD values from the experimental. This can be displayed in either of two forms: the counts of spectra as binned blocks of the RMSD of the predicted spectra from the experimental spectrum, or the cumulative sum of these counts. A solid red line indicates the position of the maximum RMSD value in the currently selected subset. Moving the cursor over the plot generates a dashed red line which moves with it displaying as it does so, the values of RMSD, count and cumulative count, allowing a user to select a new threshold RMSD value for subset selection. Clicking the left mouse button generates the new comparison position and the solid red line moves to that new position. Below this pane are detailed the experimental file name, the number of members in the current displayed subset of spectra, the matching maximum RMSD threshold for that subset, the mean RMSD of the subset, the name of the structure file within the subset from which the closest predicted spectrum to the experimental is derived, the closest RMSD of this spectrum and the structure furthest away in this subset, and its related RMSD value. To the left of this data, and below the plot, is the mean 7-state secondary structure information for the current subset as a percentage, with its associated standard deviation. The names and predicted spectra of the structures in the subset can be downloaded as a .csv file.

## CASE STUDY—UNFOLDING SIMULATIONS OF HEN EGG-WHITE LYSOZYME

To illustrate and highlight the way in which this web server might be used to provide valuable information and analyses of data, an MD simulation experiment was undertaken studying the thermal unfolding and refolding properties of Hen egg-white lysozyme (HEWL). To generate the spectra a zipped file containing the MD structures was uploaded (Figure 3A). The predicted spectra were displayed on the ‘Results’ page together with the most representative structure, along with data pertaining to the input files and secondary structure information on these structures (Figure 3B). Clustering of these predicted spectra, together with associated data on these clusters, were produced (Figure 3C) and comparisons were obtained to a series of individual experimental SRCD spectra (Figure 3D).

A similar MD study by Meersman *et al.*, (15) used pooled experimental data from SRCD spectroscopy, Fourier Transform Infra-red spectroscopy, NMR, small-angle X-ray scattering (SAXS) studies and MD simulations to examine the effects of temperature on HEWL. In this earlier study all these respective experimental techniques obtained results over the temperature range of 20–77°C, and to match this the maximum time-course for the MD runs was set at 10 nanoseconds (ns), and repeated nine times, at a temperature of 500 K. Here a different strategy was adopted; conducting MD runs as two repeats of 500 ns at temperatures of 270, 300, 350 and 450 K (full details of the MD simulation protocol are given in the Supplementary Data). Reference set structures were recorded for each trajectory; the first structure at *t* = 0, and at every 1.5 ns thereafter, such that a total of 2672 structures were produced. All these structures were pooled into one dataset. The reasoning for this strategy was that as the range of temperatures of the MD runs was broad the structures produced would be most reflective of the folded state in the lowest temperature studies, would match the partially folded states in the mid temperature runs, and would match the unfolded state in the higher temperature runs.

To ascertain the quality and relevance of the MD structures produced from our study, parameters such as secondary structure content, and radius of gyration, obtained experimentally from the other techniques reported in the original paper (15) were also generated from the structures in our MD simulations. Our criterion for MD structures to be selected as representative of the temperatures over the experimental range was solely based on the degree of matching of their predicted CD spectra to those experimental spectra reported in the SRCD data. These spectra were obtained from the PCDDB (8) resource under entry codes CD0003675000.gen to CD0003675013.gen for the unfolding spectra, and code CD0003690000.gen for the refolded from 72°C and that of the refolded 77°C data (A.J. Miles, personal communication). Each experimental SRCD spectrum from the study was compared in turn to the 2672 predicted CD spectra generated by PDBMD2CD. For each the ensemble group size of closest predicted spectra (and hence of the associated MD structures) was taken to be 60, (just over 2% of the total predicted spectra) thereby keeping the data close and relevant to the results.

Table 2 shows the comparisons between the experimental data obtained from the melting studies of lysozyme and the same parameters generated from the MD structures. The experimental CD data show lysozyme to be a very resilient structure to thermal unfolding. Little change in helix content (4%) is seen between 20 and 64°C (helix % (CD) in Table 2). Above this temperature the helix loss is more pronounced, where at 77°C the calculated content is around 19%. It is possible other forms of ‘structure’ are present at these higher temperatures which cannot be determined from the CD data. This is because there is an increase in the normalized RMSD (NRMSD) value compared to those of the lower temperature values. This term is a ‘goodness-of-fit’ between the experimental spectrum and the spectrum back-calculated using the calculated secondary structure content, which should be as close to zero as possible. The term comes from the CONTINLL (16) method that was used in DichroWeb (17) to obtain the helix secondary structure content of the experimental CD spectra. The helix content calculated from the 60 closest MD structures (Helix % (MD) in Table 2) for each of the temperatures show an excellent agreement with the CD values over the entire temperature range to 72°C. Only the 77°C values differ substantially where, as stated, the analysis of the CD data may have some issues as shown by a poor NRMSD value.

**Table 2.** Data associated with the Case Study thermal melting of Lysozyme by MD simulations in this paper and experimental data from SRCD and SAXS, obtained from ref. (15)

Temperature °C	NRMSD (CD)	RMSD (CD-MD)	Helix % (MD)	Helix % (CD)	Rg (Å) (MD)	Rg (Å) (SAX)
20	0.021	0.248	40.6 (1.3)	39 (1)	14.2 (0.1)	14.5
24	0.023	0.257	40.5 (1.2)	39 (1)	14.2 (0.1)	
28	0.024	0.262	40.5 (1.2)	39 (2)	14.2 (0.1)	
33	0.02	0.257	40.4 (1.2)	39 (2)	14.2 (0.1)	
37	0.035	0.25	40.4 (1.2)	38 (1)	14.2 (0.1)	
42	0.028	0.214	39.5 (1.4)	38 (2)	14.2 (0.1)	14.3
47	0.031	0.24	39.7 (1.4)	37 (2)	14.2 (0.1)	
51	0.035	0.203	38.6 (1.2)	37 (2)	14.2 (0.1)	
55	0.043	0.2	38.0 (1.3)	37 (1)	14.2 (0.1)	
60	0.055	0.188	37.2 (1.2)	35 (1)	14.2 (0.1)	14.2
64	0.065	0.189	35.8 (1.5)	35 (0)	14.2 (0.1)	14.3
68	0.075	0.175	33.9 (1.7)	31 (1)	14.1 (0.1)	14.3
72	0.142	0.342	21.1 (7.8)	25 (0)	15.8 (1.8)	14.9
77	0.096	0.513	7.7 (2.9)	19 (1)	16.6 (1.1)	16.6
R72*	0.037	0.215	38.1 (1.2)	36 (1)	14.2 (0.1)	
R77*	0.157	0.38	16.1 (5.7)	28 (1)	16.7 (2.1)	

*R72 and R77 refer to temperatures at 20°C having returned from 72 and 77°C.

The columns are: the temperatures of the SRCD runs (°C), the NRMSD values giving the match between the experimental CD spectrum and the back-calculated spectrum derived from the calculated secondary structure content for each temperature, the RMSD difference between the experimental CD spectra and the closest group of 60 predicted spectra (from the MD structures) at each temperature, the helix content determined from each MD group, the helix content determined from the SRCD spectra by CONTINLL method in the DichroWeb server, the radii of gyration of each group of structures, and the radii of gyration determined experimentally from low-angle X-ray scattering (SAX) studies. Standard deviations are given in brackets.

**Figure 4.**
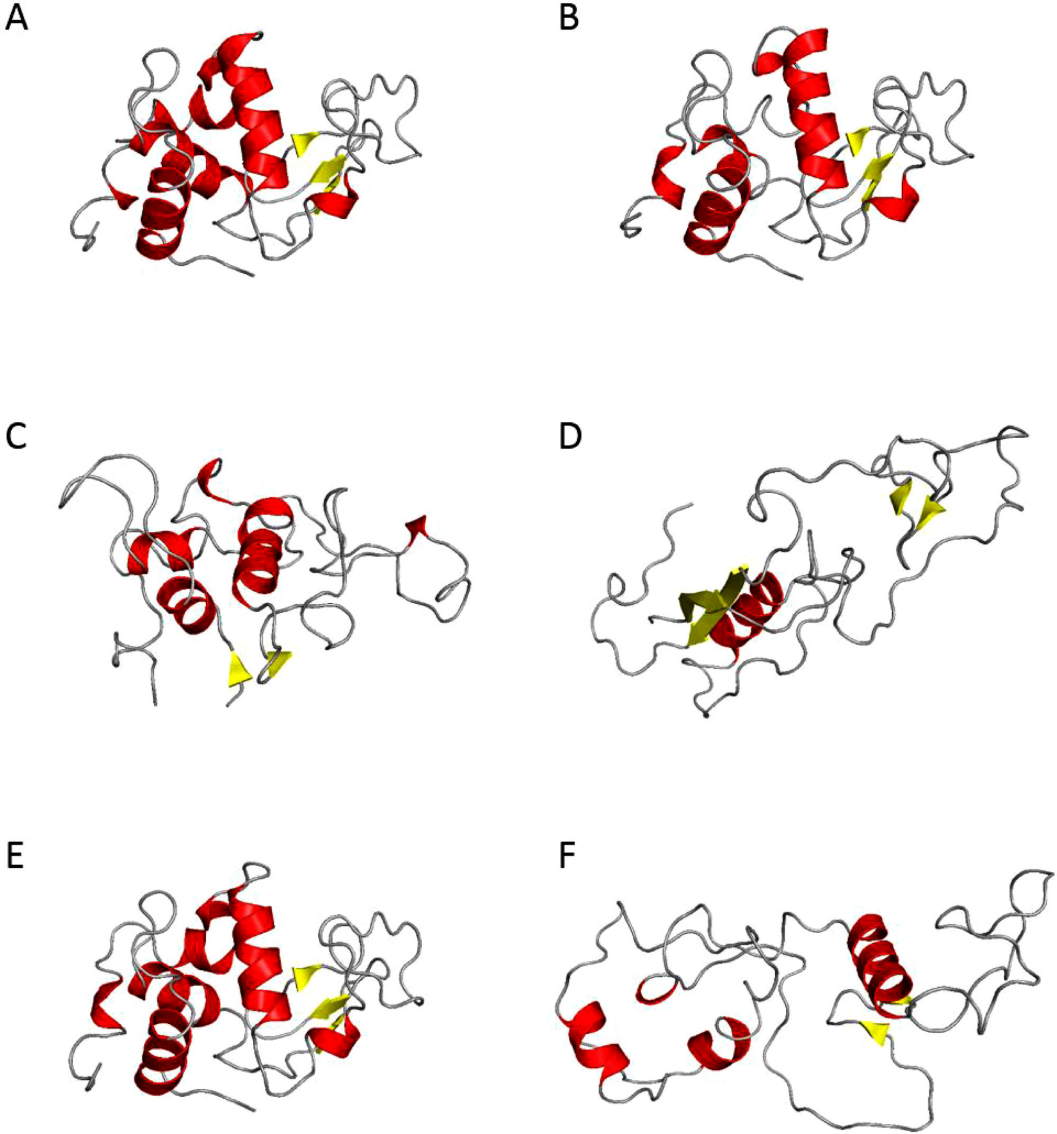
Representative structures from the MD simulation studies for the folded/unfolded states at 20, 64, 72, 77°C, and recovery to 20 from 72°C (R72) and to 20 from 77°C (R77) in panels (**A**–**F**), respectively. Where feasible, the orientations of the structures are maintained in each panel. The vertical helix in panel A is that which is retained the most in each of the subsequent panels.

Figure 4 shows representative MD simulation structures for selected temperatures from over the range of the study (the full set of structures with further associated information is given in the Supplementary Data (Supplementary Figure S2)). The radii of gyration values obtained from the SAX studies (Rg (SAXS) in Table 2) also show excellent agreement with those produced from each of these representative groups of MD structures. These indicate, again, that up to 64–68°C there is little structural change in the volume of lysozyme; i.e. the tertiary structure remains intact whilst only the ends of some helices start to unfold (as shown in Figure 4). At the highest temperature only the core of one helix and some beta sheet structure remain. Transient helix propensities are retained sufficiently at 72°C to provide a nucleus to enable recovery of the tertiary structure on cooling to quite a reasonable extent, as indicated by the data associated with the cooling back to 20°C from 72°C (R72 in Table 2). At 77°C these core helices are now lost to the point that their propensities are so low that it is not possible to recover the tertiary structure when the protein is cooled back to 20°C as indicated by the R77°C data (Table 2).

## SUMMARY

PDBMD2CD provides the user with a means of obtaining predicted CD spectra from multiple input coordinate files from a variety of sources; MD simulation structures, NMR ensemble structures and multiple homologous proteins, for example. The web server provides a ready means of interrogating predicted spectra in terms of possible clustering, offering insight into potentially different populations of structures present in the original input, or through a direct comparison to an experimental spectrum, perhaps indicating that a group of the input structures have characteristics comparable to those of the protein that produced that experimental data. As illustrated in the lysozyme case study, PDBMD2CD offers a ready means of analysing the structures from MD simulations grouping these purely by matching the degree of similarity between their predicted CD spectra to those of an experimentally-derived set of spectra from thermal unfolding and refolding studies. The helical content, and radii of gyration generated from these groups showed excellent agreement to the comparable experimentally-determined values across a wide range of temperatures. The information and analysis potential demonstrated by the PDBMD2CD web server shows that this fast and user-friendly site can provide a novel way to interrogate MD structural data.

## Supplementary Material

gkaa296_Supplemental_File

## References

[1] Mavridis L., Janes R.W. PDB2CD: a web-based application for the generation of circular dichroism spectra from protein atomic coordinates. Bioinformatics. 2017; 33:56–63. 27651482 10.1093/bioinformatics/btw554 PMC5408769

[2] Mendes L.F.S., Fontana N.A., Oliveira C.G., Freire M.C.L.C., Lopes J.L.S., Melo F.A., Costa-Filho A.J. The GRASP domain in golgi reassembly and stacking proteins: differences and similarities between lower and higher Eukaryotes. FEBS J. 2019; 286:3340–3358. 31044497 10.1111/febs.14869

[3] Caveney N.A., Pavlin A., Caballero G., Bahun M., Hodnik V., de Castro L., Fornelos N., Butala M., Strynadka N.C.J. Structural insights into bacteriophage GIL01 gp7 inhibition of host LexA repressor. Structure. 2019; 27:1094–1102. 31056420 10.1016/j.str.2019.03.019

[4] Osterlund N., Kulkarni Y.S., Misiaszek A.D., Wallin C., Kruger D.M., Liao Q.H., Rad F.M., Jarvet J., Strodel B., Warmlander S.K.T.S. Amyloid-β Peptide interactions with amphiphilic surfactants: electrostatic and hydrophobic effects. ACS Chem. Neurosci. 2018; 9:1680–1692. 29683649 10.1021/acschemneuro.8b00065

[5] Wang F., Orioli S., Ianeselli A., Spagnolli G., Beccara S.A., Gershenson A., Faccioli P., Wintrode P.L. All-atom simulations reveal how single-point mutations promote serpin misfolding. Biophys. J. 2018; 114:2083–2094. 29742402 10.1016/j.bpj.2018.03.027 PMC5961751

[6] Zheng X., Mueller G.A., Kim K., Perera P., Eugene F., DeRose E.F., London R.E. Identification of drivers for the metamorphic transition of HIV-1 reverse transcriptase. Biochem. J. 2017; 474:3321–3338. 28811321 10.1042/BCJ20170480 PMC5614878

[7] Lees J.G., Miles A.J., Wien F., Wallace B.A. A reference database for circular dichroism spectroscopy covering fold and secondary structure space. Bioinformatics. 2006; 22:1955–1962. 16787970 10.1093/bioinformatics/btl327

[8] Whitmore L., Miles A.J., Mavridis L., Janes R.W., Wallace B.A. PCDDB: new developments at the Protein Circular Dichroism Data Bank. Nucleic Acids Res. 2017; 45:D303–D307. 27613420 10.1093/nar/gkw796 PMC5210608

[9] Abdul-Gader A., Miles A.J., Wallace B.A. A reference dataset for the analyses of membrane protein secondary structures and transmembrane residues using circular dichroism spectroscopy. Bioinformatics. 2011; 27:1630–1636. 21505036 10.1093/bioinformatics/btr234

[10] Jasim S.B., Li Z., Guest E.E., Hirst J.D. DichroCalc: improvements in computing protein circular dichroism spectroscopy in the near-ultraviolet. J. Mol. Biol. 2018; 430:2196–2202. 29258819 10.1016/j.jmb.2017.12.009

[11] Nagy G., Igaev M., Jones N.C., Hoffmann S.V., Grubmüller H. SESCA: predicting circular dichroism spectra from protein molecular structures. J. Chem. Theory Comput. 2019; 15:5087–5102. 31402660 10.1021/acs.jctc.9b00203

[12] Berman H.M., Westbrook J., Feng Z., Gilliland G., Bhat T.N., Weissig H., Shindyalov I.N., Bourne P.E. The Protein Data Bank. Nucleic Acids Res. 2000; 28:235–242. 10592235 10.1093/nar/28.1.235 PMC102472

[13] Kabsch W., Sander C. Dictionary of protein secondary structure: pattern recognition of hydrogen-bonded and geometrical features. Biopolymers. 1983; 22:2577–2637. 6667333 10.1002/bip.360221211

[14] MacQueen J. Some methods for classification and analysis of multivariate observations. Proceedings of the Fifth Berkeley Symposium on Mathematical Statistics and Probability. 1967; 1:281–297.

[15] Meersman F., Atilgan C., Miles A.J., Bader R., Shang W.F., Matagne A., Wallace B.A., Koch M.H.J. Consistent picture of the reversible thermal unfolding of hen egg-white lysozyme from experiment and molecular dynamics. Biophys. J. 2010; 99:2255–2263. 20923660 10.1016/j.bpj.2010.07.060 PMC3042584

[16] Van Stokkum I.H.M., Spoelder H.J.W., Bloemendal M., Van Grondelle R., Groen F.C.A. Estimation of protein secondary structure and error analysis from CD spectra. Anal. Biochem. 1990; 191:110–118. 2077933 10.1016/0003-2697(90)90396-q

[17] Whitmore L., Wallace B.A. Protein secondary structure analyses from circular dichroism spectroscopy: methods and reference databases. Biopolymers. 2008; 89:392–400. 17896349 10.1002/bip.20853

[18] Micsonai A., Wien F., Bulyáki E., Kun J., Moussong E., Lee Y.H., Goto Y., Réfrégiers M., Kardos J. Accurate secondary structure prediction and fold recognition for circular dichroism spectroscopy. Proc. Natl. Acad. Sci. U.S.A. 2015; 112:E3095–E3103. 26038575 10.1073/pnas.1500851112 PMC4475991

