# Supplementary material for: PDBMD2CD: providing predicted protein circular dichroism spectra from multiple molecular dynamics-generated protein structures
Source: Nucleic Acids Res. 2020 Apr 28;48(W1):W17–24. doi: 10.1093/nar/gkaa296 (PMC7319452; doi:10.1093/nar/gkaa296)
Supplement: gkaa296_Supplemental_File [file gkaa296_supplemental_file.pdf]

PDBMD2CD: Providing Predicted Protein Circular Dichroism Spectra from Multiple Molecular Dynamics-Generated Protein Structures

Elliot D. Drew and Robert W. Janes*

School of Biological and Chemical Sciences, Queen Mary University of London, Mile End Road, London, E1 4NS, UK

*To whom correspondence should be addressed. Tel: +44 207 8828442; Email: r.w.janes@qmul.ac.uk

Supplementary Data

METHODS AND MATERIALS

Reference Dataset

The reference data set consists of 83 proteins (Table S1) selected from the SMP180 "golden standard" SRCD spectral data set (1), used in the DichroWeb deconvolution server (2) and available at the PCDDB (3). All proteins in SMP180 have well-determined SRCD spectra as reported by ValiDichro (4) and have associated PDB structures, and includes the SP175 (5) data set. Proteins with spectral characteristics arising from non-secondary structural phenomena, (for example ligands, or possible exciton-coupling of aromatic side chains), were removed from the data set as these features led to inaccuracy in the predictions produced. In addition, proteins whose removal from the reference set resulted in an increased accuracy during leave-one-out cross validation were ultimately excluded from the 83 proteins of the final reference set. The reasoning for this was that their inclusion in the training set would lead to a decrease in prediction performance for the majority of proteins.

Test data

Eight proteins were identified in the PCDDB which satisfied the criteria of having well-determined CD spectra, as shown by their ValiDichro reports, and having associated PDB structures (Table S3). These were used as a wholly independent further test set for the PDBMD2CD method.

Input to the server

The server accepts both PDB/mmCIF structure files and PDB codes. For analysis of PDB structure files, single files or multiple files can be uploaded. Multiple files can also be uploaded as a zip, bzip or tar.gz archive file. For PDB codes, multiple files can be submitted by separating codes with a comma.

The server has been tested with >1000 structures in a single job, which yielded a result (not including time to upload) in under 5 minutes, making it suitable for MD trajectory analysis.

Secondary structure assignment

The 8 state secondary structure assignments of input structures are calculated by DSSP (6). In Table S2 the mapping between our classification and DSSP classes is shown. Additionally, information about missing residues in the structure (those present in the sample but whose positions could not be assigned often due to intrinsic disorder or high flexibility), is obtained, if present from the header of the structure file. The number of missing residues found is added to the C (considered as "O" here for "Other") DSSP class. The assignments for Beta strand ("E") are further processed to produce the final assignments used in the method.

The CD signals produced by beta-sheet structures are diverse due to the variety of topological arrangements possible in this class. Strands can be arranged in antiparallel or parallel sheets and these can display varying degrees of distortion or twist. Manavalan and Johnson (7) observed that beta-rich proteins fall into two classes with respect to their CD spectra: one class has a "classical" beta-sheet spectrum (negative band ~218 nm, positive band ~195 nm); the other class has a CD spectrum more like that of unordered proteins (negative band near 200 nm). Wu et al. (8) designated these two classes as beta-I and beta-II, respectively. More recently, a treatment of protein twist has been incorporated into the secondary structure determination tool BeStSel (9).

Given the strong correlations between antiparallel sheet distortion and CD spectrum shape (Fig S1), residues assigned as "E" by DSSP are partitioned into three separate classes - P for parallel sheets, AP1 for sheets with minimal distortion and AP2 for "distorted" sheets. Parallel or antiparallel status is determined from information in the DSSP output. Classification of AP1 and AP2 is more complex - in a beta sheet, a residue might interact through hydrogen bonding with multiple residues located in strands N- and C- terminal to its own strand. It might be in a distorted arrangement with one, both or none of its interacting partners. Therefore, we calculate the number of non-distorted vs distorted interactions and apply that ratio to the total count of anti-parallel residues to obtain a final count for the AP1 and AP2 classes.

To determine if a residue, *i*, is part of a distorted strand interaction, the sheet hydrogen bonding network of all anti-parallel residues in the protein is extracted from the DSSP output. The distance and direction between the Cα atom of residue *i* and the Cα atom of the residue N-terminal to *i* in the strand is calculated and stored as a vector as nodes in the network. Edges for node *i* are made to nodes corresponding to residues that form a hydrogen bond with *i.*

All edges in the network are then iterated through and the angle θ between the two vectors *x* and *y* associated with connected nodes, corresponding to the local geometry of their respective strands, is calculated using the dot product:

$$\theta = \cos^{-1}\left(\frac{x \cdot y}{||\mathrm{x}||\ ||\mathrm{y}||}\right)$$

With a θ in the range 35° - 110° the residue is considered to be engaging in a distorted interaction with its partner, and we add one to the count of distorted interactions. Once all interactions have been assessed, we convert the count into a fraction by dividing by the total number of edges in the network. We determine the final total for AP1 and AP2 as below:

$$AP1 = AP - AP2$$
$$AP2 = AP \cdot f_{dist}$$

Where AP is the total number of anti-parallel residues and f_{dist} is the fraction of distorted interactions observed.

Prediction of spectra

Two separate models are used to predict the final spectrum, detailed below.

Least squares model

The secondary structure assignments from DSSP are modified to produce a seven state description of secondary structure, by combining the I ("π helix"), S ("Bend") and B ("isolated β Bridge") assignments into the O class. This results in H1, H2 ("3_{10} helix"), A1, A2, P, T and O. Linear least-squares regression using the secondary structure assignments and circular dichroism spectra of the reference proteins were used to build a model for each wavelength from 180 nm to 260 nm. The *m* CD spectra of all proteins in the reference data set, covering *n* observations at wavelengths in the previously stated range, were stored as an *m* by *n* matrix. This matrix was then transposed to obtain an *n* by *m* matrix with each row containing the CD signal for each protein at a specific wavelength. The secondary structure predictions for each protein were stored as an *m* by 7 matrix. The model weightings (*x*) for each wavelength *n* were obtained by solving the equation *a x* = *b* by computing a vector *x* that minimizes the Euclidean norm, L^2:

$$L^2 = ||b - ax||^2$$

where *a* is the *m* length vector of CD data points at wavelength *n* and *b* is the matrix containing the secondary structure predictions. From these models, seven basis spectra were calculated by multiplying the weightings *x* at each wavelength *n* by a 7-length vector corresponding to 100% of each secondary structure type in turn.

The prediction is made by multiplying the basis spectra by the corresponding fraction of secondary structure states determined for the query protein by DSSP and summing the resulting spectra.

Linear Combination method

For this method, an eight-state secondary structure assignment is used, adding the DSSP B and S class to the O class, to generate H1, H2, AP1, AP2, P, I, T and O. The 12 proteins with the closest

secondary structure content to the query are selected from the reference data set to act as basis spectra for the prediction. The equation below is then solved using the Broyden–Fletcher–Goldfarb–Shanno (BFGS) optimisation algorithm implemented in the SciPy python package (10) to find the vector *x* of length *n* corresponding to the weightings applied to the *m* by 8 matrix of the basis protein's secondary structure, b_{pred}, to best fit the secondary structure vector of the query, b_{query}.

$$b_{query} \approx x \cdot b_{pred}$$

The product of the calculated weightings and CD spectra of the *m* basis set proteins results in the predicted CD spectrum of the query.

Calculation of final predicted spectrum.

The spectra resulting from the least squares model and the linear combination method are combined through averaging. The averaged spectrum that results led to better overall accuracy on our reference set (0.996 (0.41)) than either method in isolation (least squares - 0.977 (0.39) Δε, linear combination - 0.996 (0.41) Δε).

Method validation

Leave-one-out cross validation on the training reference data set was used to provide insight on how the method would generalise to an unknown data set. A separate validation was performed on an independent test set consisting of 8 proteins (see Test Set). In all cases the root mean squared deviation (RMSD) of the experimental spectrum versus the predicted spectrum was used to assess accuracy.

The RMSD was calculated using the following equation:

$$RMSD = \sqrt{\frac{\sum_{i=1}^{N}(e_i - p_i)^2}{N}}$$

Where *N* is the number of data points, e_i is the experimental CD signal at each wavelength *i* and p_i is the predicted CD signal at each wavelength *i*.

K-means analysis tool

If the number of predictions made is >=50, the data are partitioned using the k-means clustering algorithm implemented in the SciPy python packaged (10). k-Means clustering partitions observations into k clusters (where k is defined beforehand) so each observation belongs to the cluster with the nearest mean, by minimising the within cluster variances. For k=1 to k=6 inclusive, calculations are performed such that all predicted spectra are clustered using the Euclidean distance between spectra. As the user chooses the value of k, from a dropdown menu, the clustering page automatically updates

the information shown with the pre-calculated information. The mean spectrum, the representative structure (defined as the structure with predicted spectrum with lowest RMSD to the calculated mean) and the average secondary structure values of each cluster at each value of k are obtained. These are presented to the user in the "Clustering" tab of the results page.

An elbow plot showing k versus the distortion is provided to guide the user in the choice of an appropriate value of k. The distortion is calculated as the mean (non-squared) Euclidean distance between the observations passed and the centroids generated for a given value of k. As a general rule of thumb, the optimal value k will be at the "elbow" i.e. the point after which the distortions begin to decrease in a linear fashion.

"Comparison with experiment" tool

The webserver provides a facility to compare a prediction or set of predictions against an uploaded experimental spectrum. Based on user-defined parameters, the tool will produce a subset of predictions closest to the experimental spectra and provide summary statistics on said set. The uploaded spectrum can be in units of Delta Epsilon or Mean Residue Ellipticity (MRE) - selection of the appropriate option on the results page before upload will automatically convert MRE values to delta epsilon using the following equation:

$$\Delta\varepsilon = \mathrm{MRE}/3298$$

The RMSD between all predictions and the experimental spectrum is calculated and the values are then sorted from smallest to largest. There are two options available to the user, choosing subsets of predictions using either a maximum RMSD from experimental spectrum threshold, or by defining a maximum number of predictions, N, to retrieve. Using the former, all predictions with RMSD less than or equal to this value are collected and presented to the user as a set. Using the latter, the N predictions closest to the experimental (as determined by RMSD) forms the set presented to the user.

A range of statistics about the subset are presented to the user, including average secondary structure percentages; RMSD of closest prediction to experimental; RMSD of subset average prediction to the experimental; number of members in the subset; etc. A downloadable .csv file containing the predicted spectra and names of all structures in the set is available to facilitate further analysis by the user.

Cast Study - Unfolding Simulations of Hen Egg White lysozyme

The structure of Hen Egg White lysozyme (HEWL) was obtained from the PDB (PDBID: 2VB1). The CHARMM-GUI webserver (11, 12) was used to generate input for simulations at 270K, 300K, 350K and 450K. All simulations were initiated with 2VB1 as their starting structure. The starting structure was solvated in TIP3 water with potassium and sodium ions added to neutralise the charge of the system. Minimisation, equilibration and production runs were carried out using GROMACS 2019 (13). Minimisation was accomplished using the steepest descent algorithm for 5000 steps. All systems were equilibrated at their specific temperatures for 2.5 ns with a 1 fs timestep using the Nose-Hoover thermostat. Production runs were carried out for 500 ns with a 2 fs timestep using the Nose-Hoover

thermostat and Parrinello-Rahman pressure coupling. At each temperature, two simulations were performed yielding 4 µs of total simulation time across all 8 simulations.

Analysis of MD simulations and comparison with experimental data

Frames were extracted every 1.5 ns from the 8 production runs and saved as PDB formatted files, for a total of 2672 structures that comprised the pool for PDBMD2CD. DSSP was used to obtain the per-residue secondary structure assignments and total secondary structure class count for each structure. The gmx gyrate tool included with GROMACS was used to obtain radius of gyration values for all structures. Predicted spectra for each structure were obtained using the PDBMD2CD webserver.

In vitro experimental CD spectra of HEWL were obtained between 20 ^{O}C and 77 ^{O}C from their PCDDB entries (main text) as was the refold spectrum to 20 ^{O}C from 72 ^{O}C (R72) while data for the 77 ^{O}C (R77) was provided by Dr A.J. Miles (personal communication). Radius of gyration values from Small Angle X-Ray Scattering (SAXS) experiments between 20 ^{O}C and 80 ^{O}C were obtained from data in Meersman et al. (14).

For each experimental CD spectrum, the 60 closest representative structures were obtained using the "Comparison with experiment" tool on the PDBMD2CD website based on the RMSD between the experimental spectra and the predictions. The list of protein structures and their associated predicted spectra were downloaded from the site. Average DSSP secondary structure assignments, per-residue helical propensities and average radius of gyration were calculated for each representative set, and compared against the corresponding data reported in Meersman et al. (14) for the given experimental temperature.

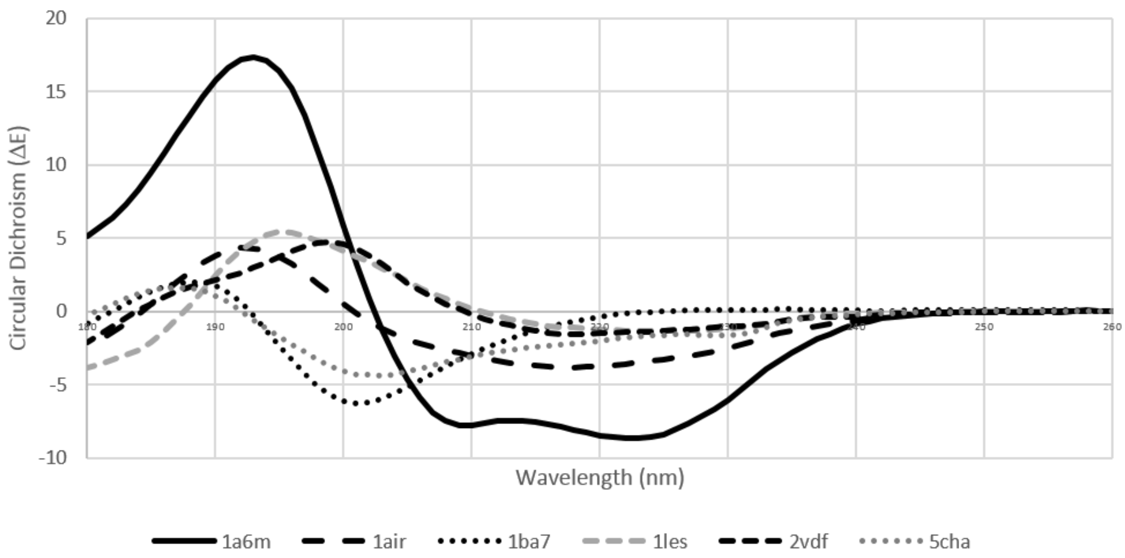

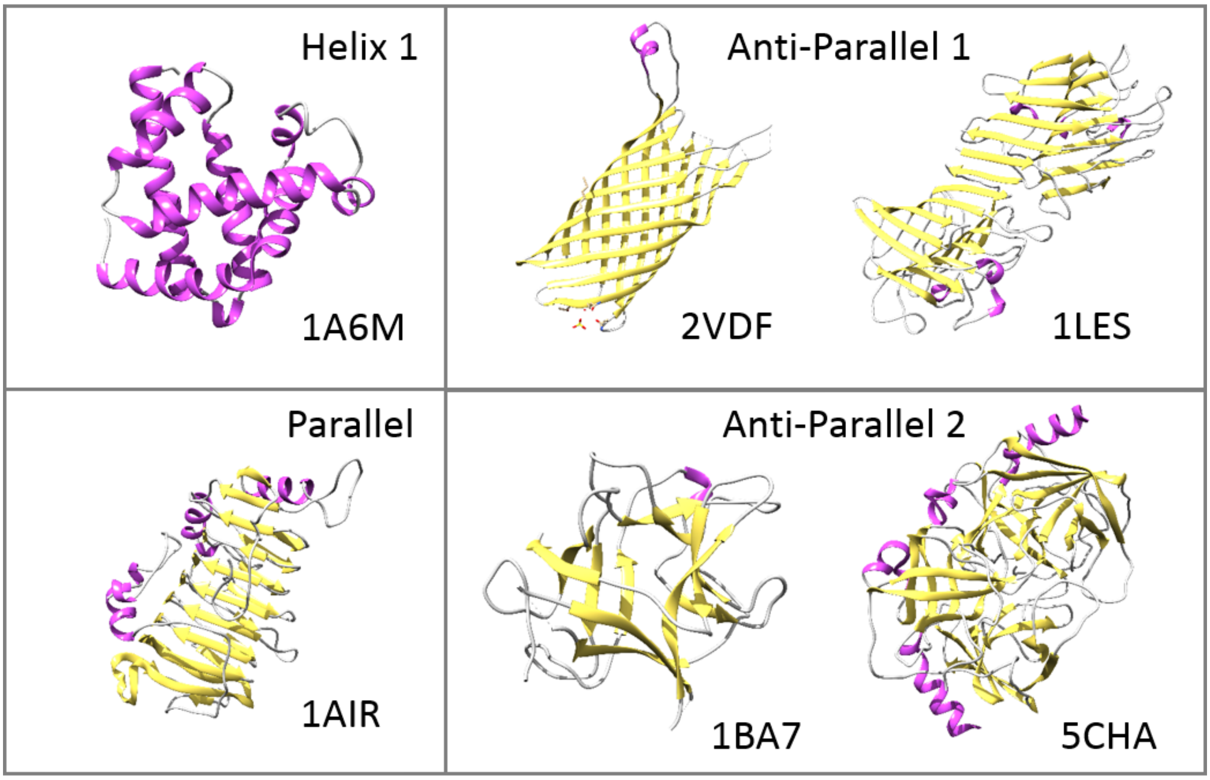

PDB ID<br>PCDDBID	1a6m<br>CD0000048000	1air<br>CD0000054000	1ba7<br>CD0000065000	1les<br>CD0000043000	2vdf<br>CD0000119000	5cha<br>CD0000005000
**Helix 1**	0.74	0.09	0.00	0.02	0.02	0.09
**Helix 2**	0.04	0.04	0.02	0.03	0.00	0.03
**Anti-Parallel Strand 1**	0.00	0.00	0.15	0.39	0.59	0.16
**Anti-Parallel Strand 2**	0.00	0.01	0.21	0.08	0.13	0.15
**Parallel Strand**	0.00	0.31	0.00	0.01	0.02	0.02
**Turn**	0.09	0.08	0.11	0.11	0.03	0.12
**Other**	0.13	0.47	0.51	0.37	0.21	0.44

Figure S1: Secondary structure content including beta distortion defines CD spectral characteristics. Presented are the CD spectra, 3D structures and 7 state PDBMD2CD secondary structure classification for six proteins. These act as exemplars for Helix 1 (1A6M – sperm-whale myoglobin), Anti-parallel 1 (minimal distorted) (2VDF - OpcA adhesion protein; 1LES – lentil lectin), Anti-parallel 2 (distorted sheet) (1BA7 - soybean trypsin inhibitor; 5CHA – alpha-chymotrypsin) and parallel (1AIR – pectate lyase C). There is a clear difference between a high content AP1 spectrum which have maxima between ~190 nm and ~200 nm and a high content AP2 spectrum, with a maxima ~186 nm and minima ~202nm. This difference is correlated with the fraction of anti-parallel beta sheet residues involved in "distorted" interactions with their inter-strand hydrogen bonding partners, that has also been noted by others (9).

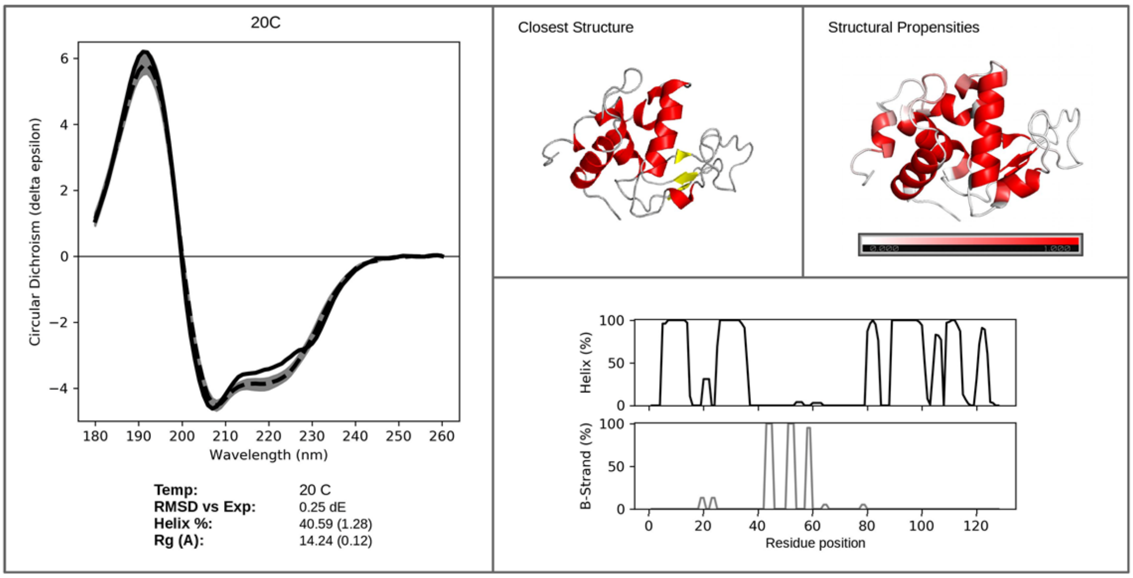

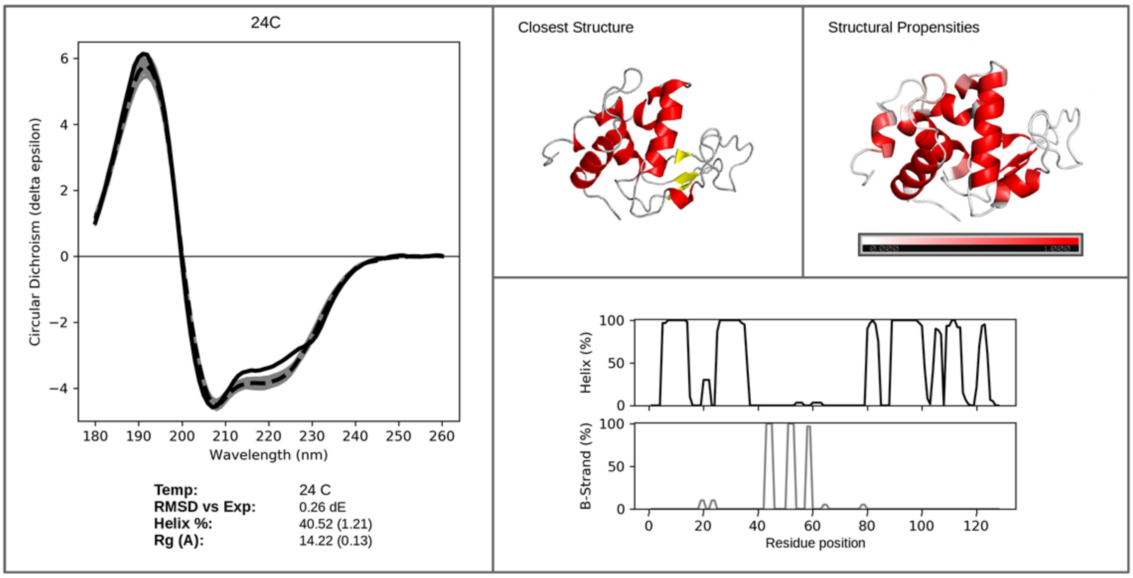

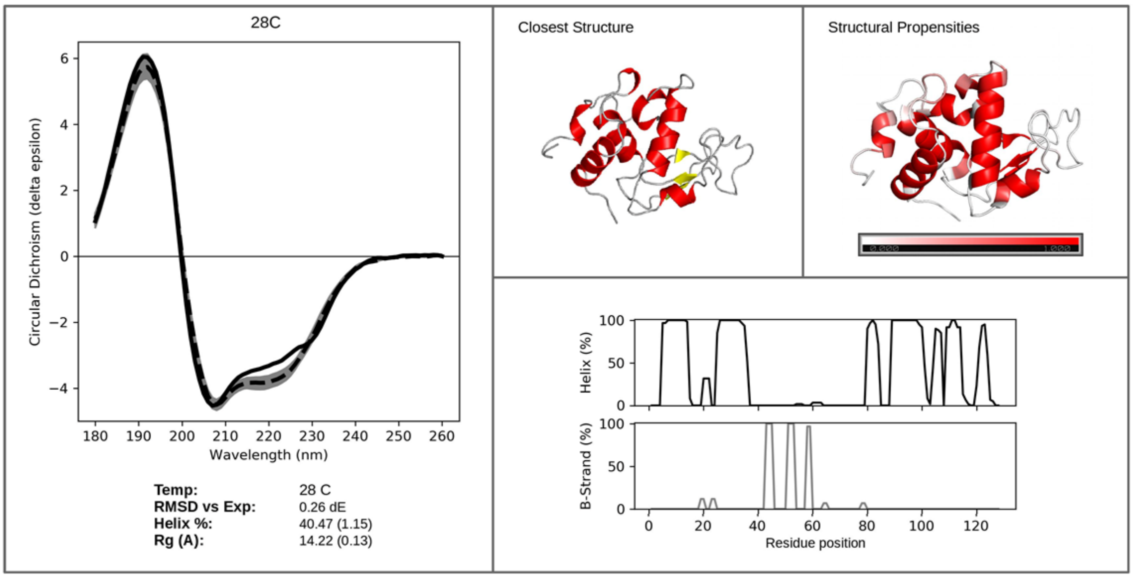
28C
Circular Dichroism (delta epsilon)
Wavelength (nm)
Temp: 28 C
RMSD vs Exp: 0.26 dE
Helix %: 40.47 (1.15)
Rg (A): 14.22 (0.13)
Closest Structure
Structural Propensities
Helix (%)
B-Strand (%)
Residue position

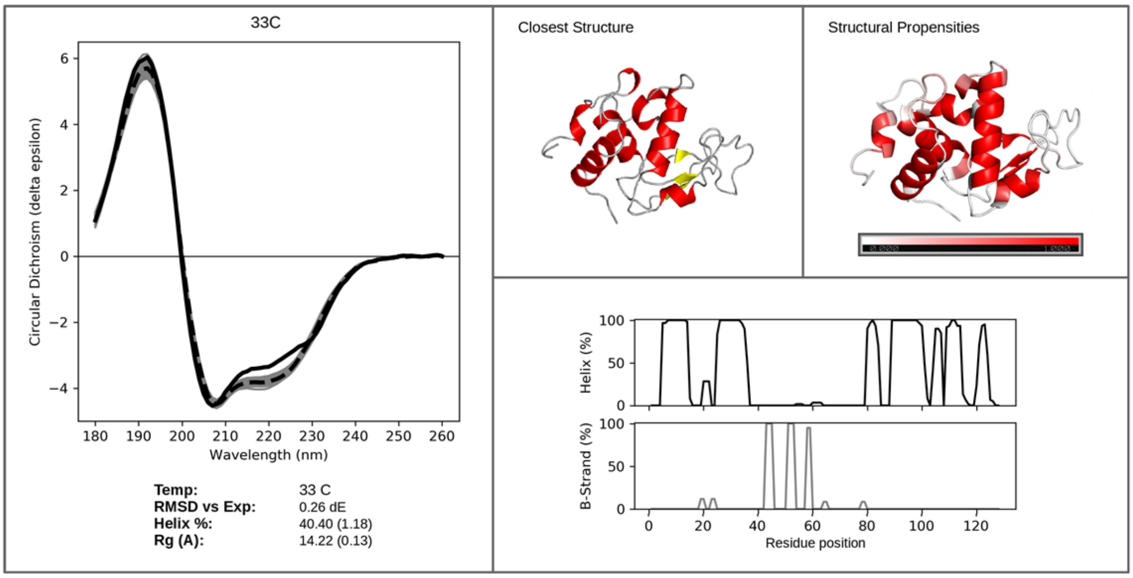
33C
Circular Dichroism (delta epsilon)
Wavelength (nm)
Temp: 33 C
RMSD vs Exp: 0.26 dE
Helix %: 40.40 (1.18)
Rg (A): 14.22 (0.13)
Closest Structure
Structural Propensities
Helix (%)
B-Strand (%)
Residue position

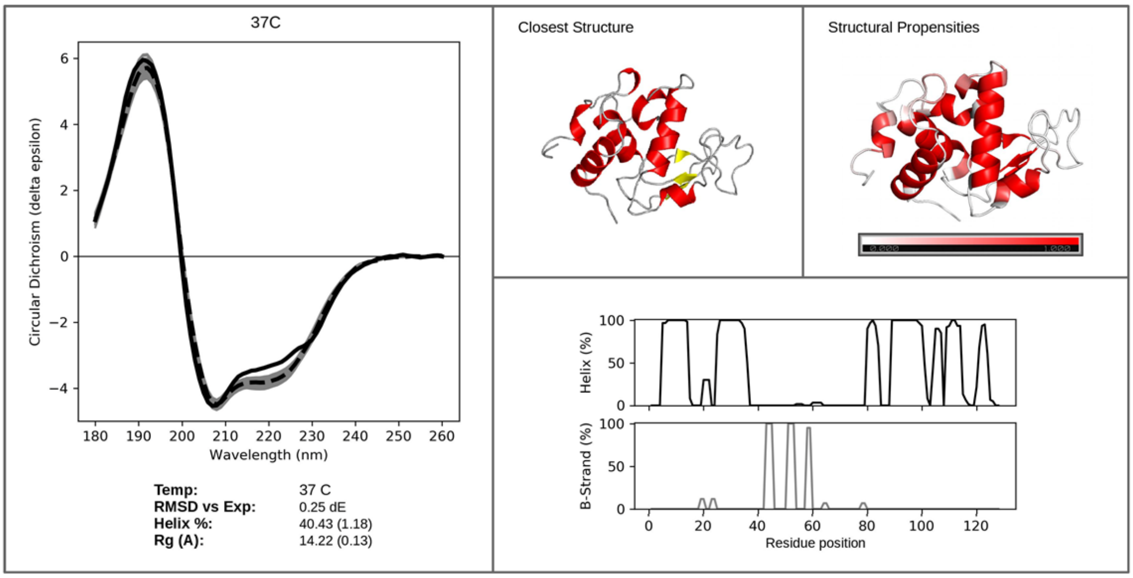
37C
Circular Dichroism (delta epsilon)
Wavelength (nm)
Temp: 37 C
RMSD vs Exp: 0.25 dE
Helix %: 40.43 (1.18)
Rg (A): 14.22 (0.13)
Closest Structure
Structural Propensities
Helix (%)
B-Strand (%)
Residue position

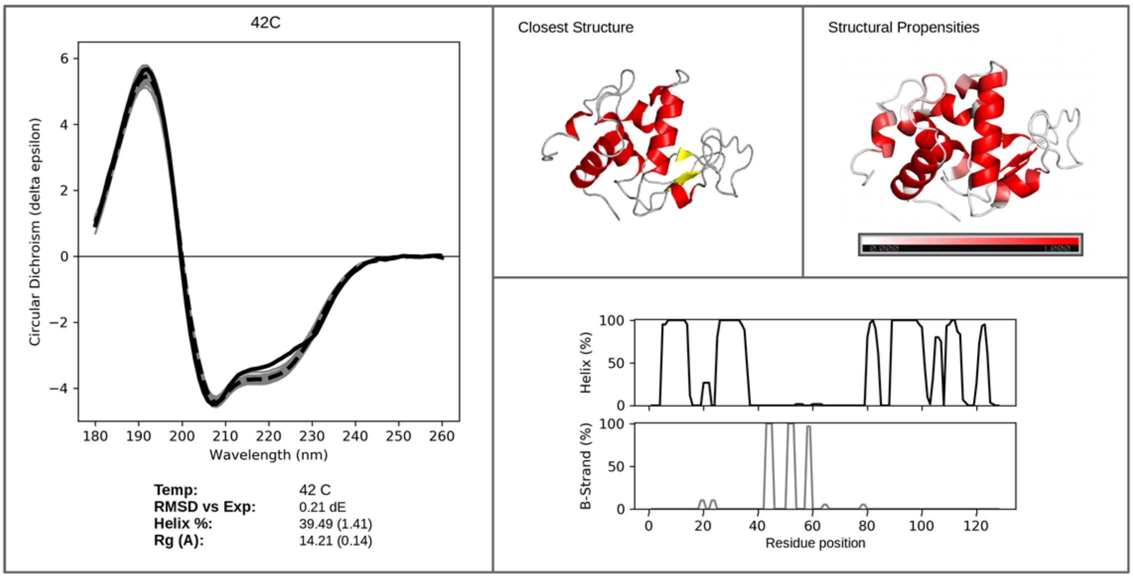
42C
Circular Dichroism (delta epsilon)
Wavelength (nm)
Temp: 42 C
RMSD vs Exp: 0.21 dE
Helix %: 39.49 (1.41)
Rg (A): 14.21 (0.14)
Closest Structure
Structural Propensities
Helix (%)
B-Strand (%)
Residue position

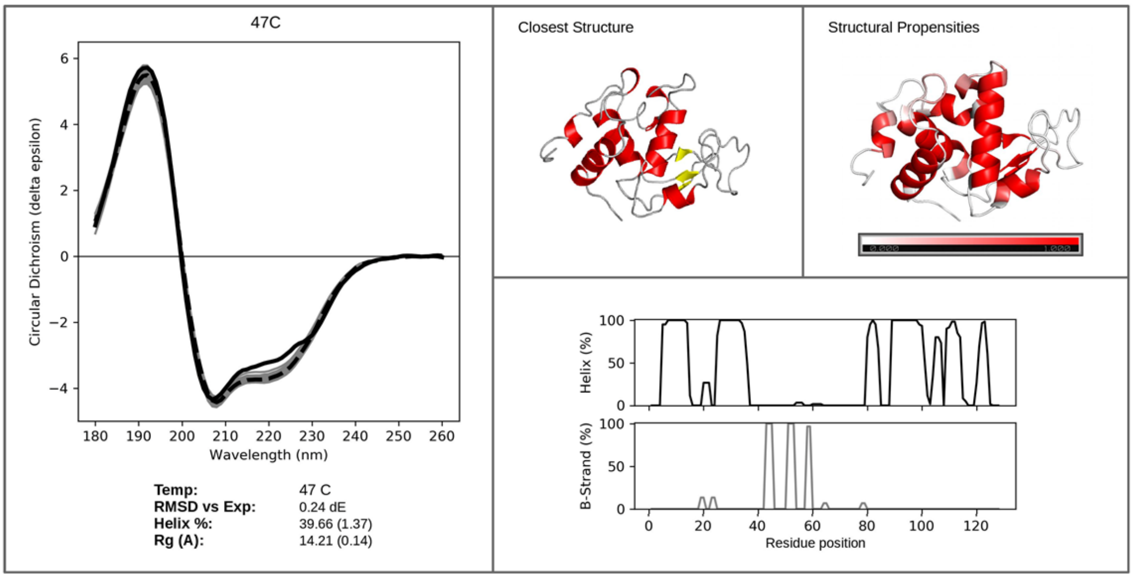
47C
Circular Dichroism (delta epsilon)
Wavelength (nm)
Closest Structure
Structural Propensities
Helix (%)
B-Strand (%)
Residue position
Temp: 47 C
RMSD vs Exp: 0.24 dE
Helix %: 39.66 (1.37)
Rg (A): 14.21 (0.14)

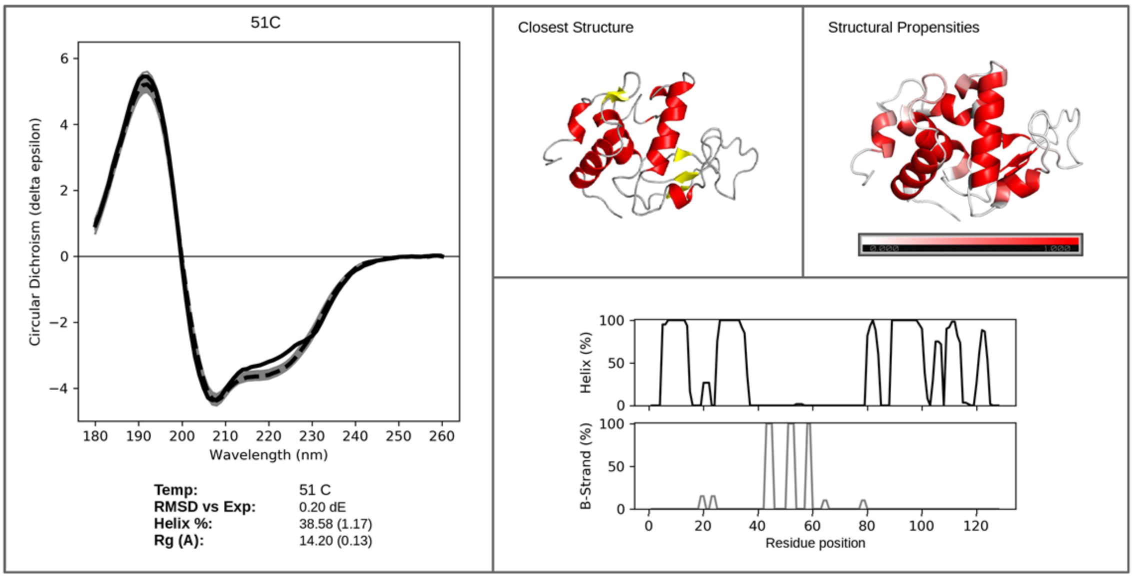
51C
Circular Dichroism (delta epsilon)
Wavelength (nm)
Closest Structure
Structural Propensities
Helix (%)
B-Strand (%)
Residue position
Temp: 51 C
RMSD vs Exp: 0.20 dE
Helix %: 38.58 (1.17)
Rg (A): 14.20 (0.13)

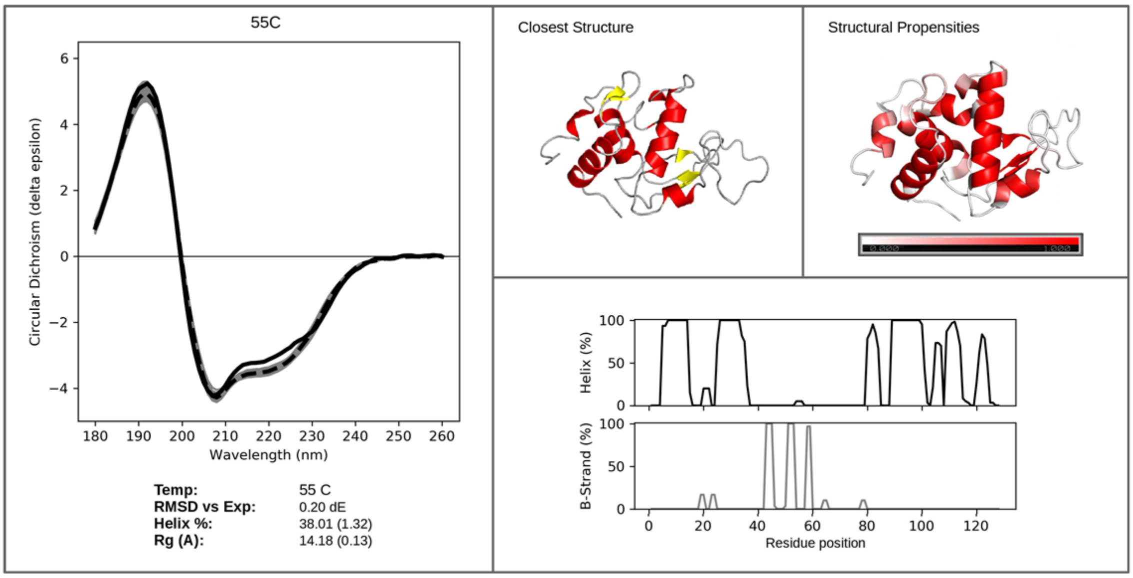
55C
Circular Dichroism (delta epsilon)
Wavelength (nm)
Temp: 55 C
RMSD vs Exp: 0.20 dE
Helix %: 38.01 (1.32)
Rg (A): 14.18 (0.13)
Closest Structure
Structural Propensities
Helix (%)
B-Strand (%)
Residue position

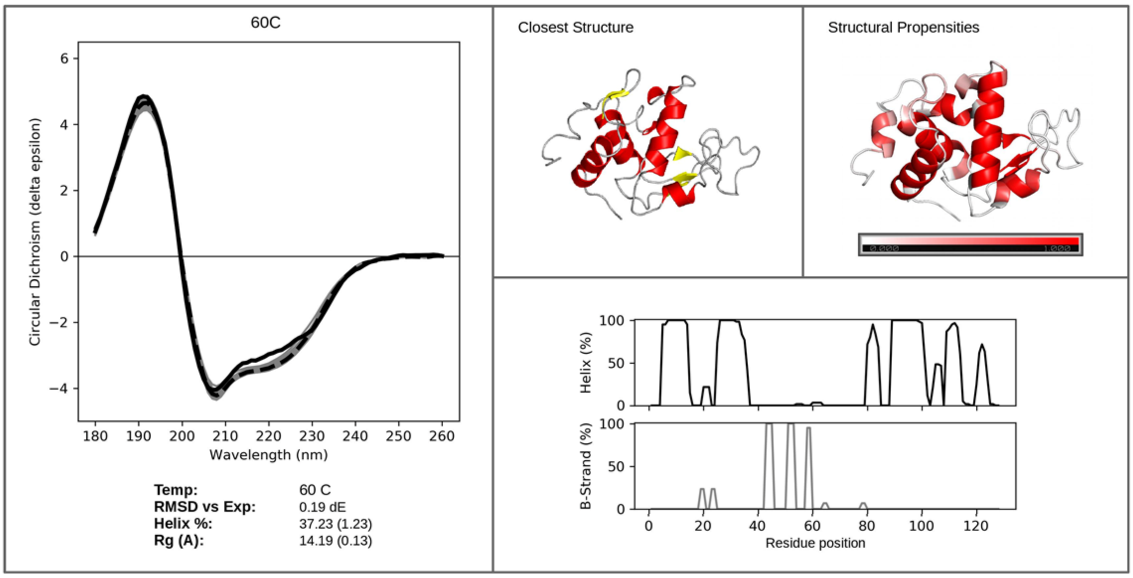
60C
Circular Dichroism (delta epsilon)
Wavelength (nm)
Temp: 60 C
RMSD vs Exp: 0.19 dE
Helix %: 37.23 (1.23)
Rg (A): 14.19 (0.13)
Closest Structure
Structural Propensities
Helix (%)
B-Strand (%)
Residue position

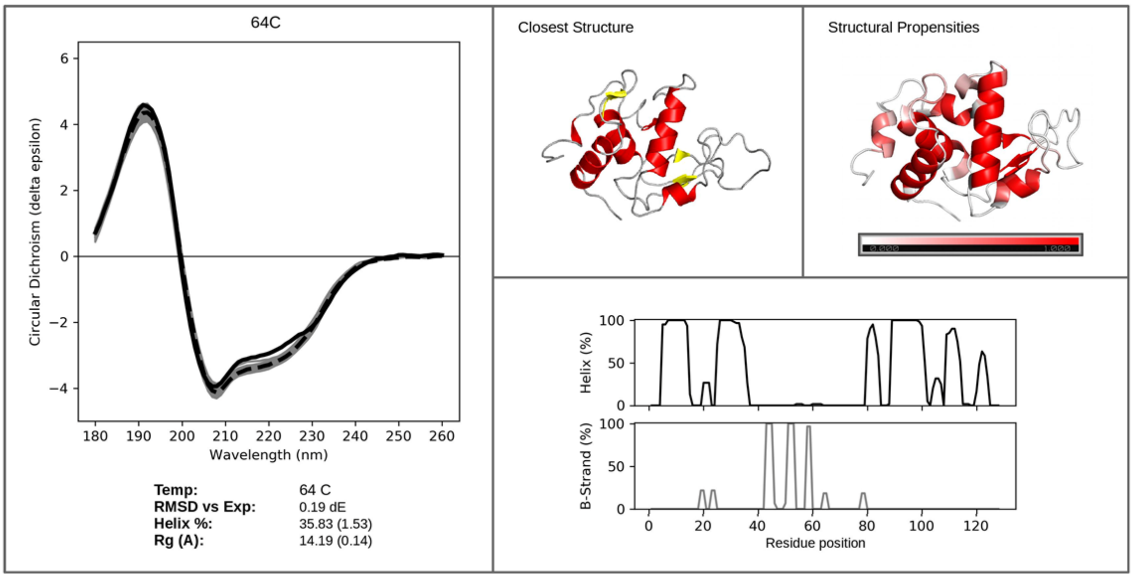
64C
Circular Dichroism (delta epsilon)
Wavelength (nm)
Temp: 64 C
RMSD vs Exp: 0.19 dE
Helix %: 35.83 (1.53)
Rg (A): 14.19 (0.14)
Closest Structure
Structural Propensities
Helix (%)
B-Strand (%)
Residue position

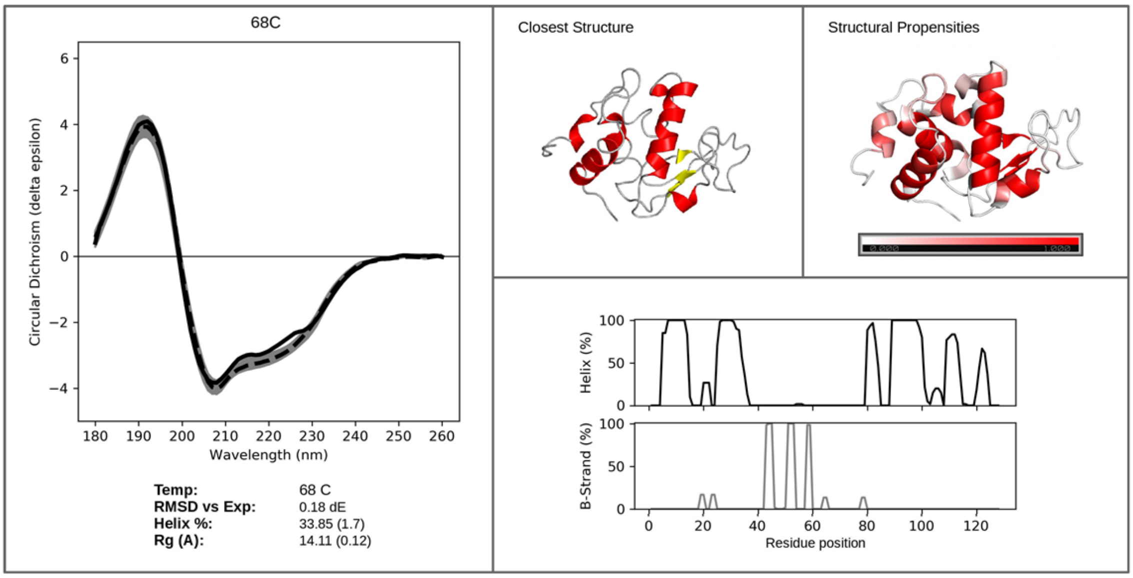
68C
Circular Dichroism (delta epsilon)
Wavelength (nm)
Temp: 68 C
RMSD vs Exp: 0.18 dE
Helix %: 33.85 (1.7)
Rg (A): 14.11 (0.12)
Closest Structure
Structural Propensities
Helix (%)
B-Strand (%)
Residue position

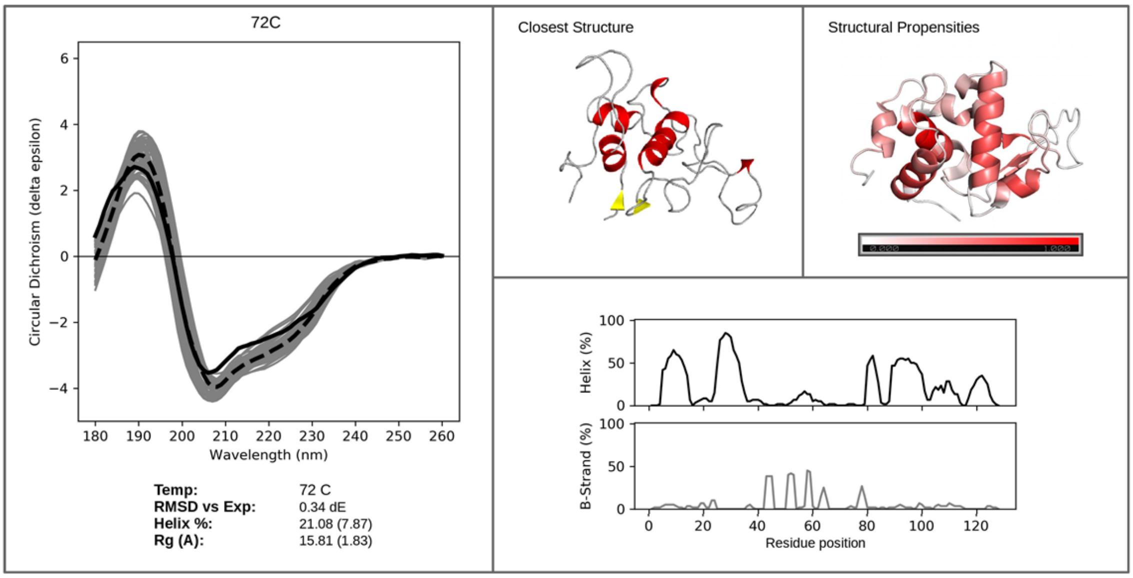
72C
Circular Dichroism (delta epsilon)
Wavelength (nm)
Temp: 72 C
RMSD vs Exp: 0.34 dE
Helix %: 21.08 (7.87)
Rg (A): 15.81 (1.83)
Closest Structure
Structural Propensities
Helix (%)
B-Strand (%)
Residue position

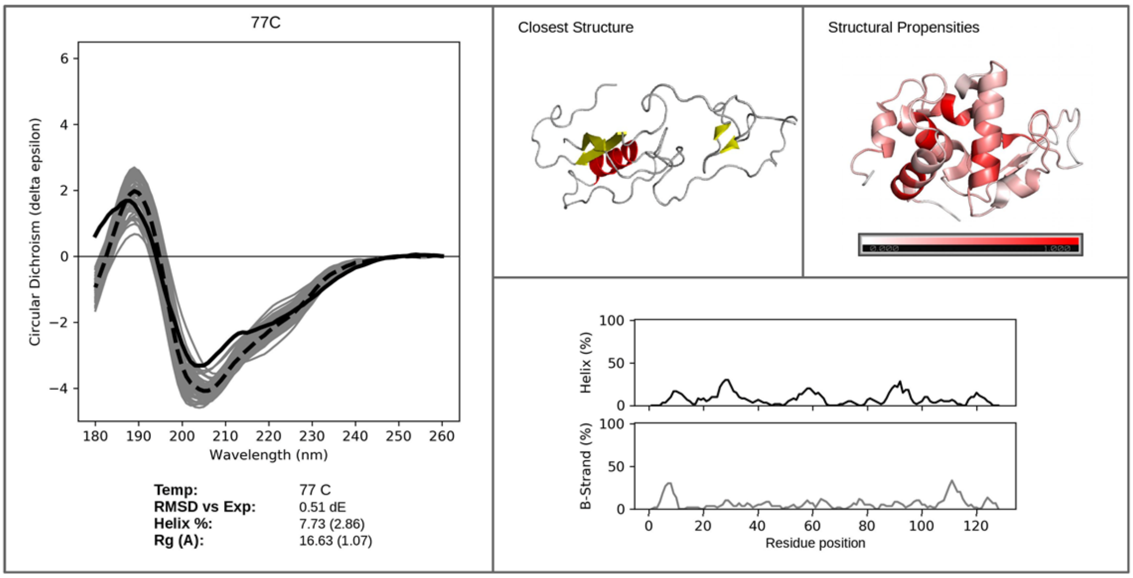
77C
Circular Dichroism (delta epsilon)
Wavelength (nm)
Temp: 77 C
RMSD vs Exp: 0.51 dE
Helix %: 7.73 (2.86)
Rg (A): 16.63 (1.07)
Closest Structure
Structural Propensities
Helix (%)
B-Strand (%)
Residue position

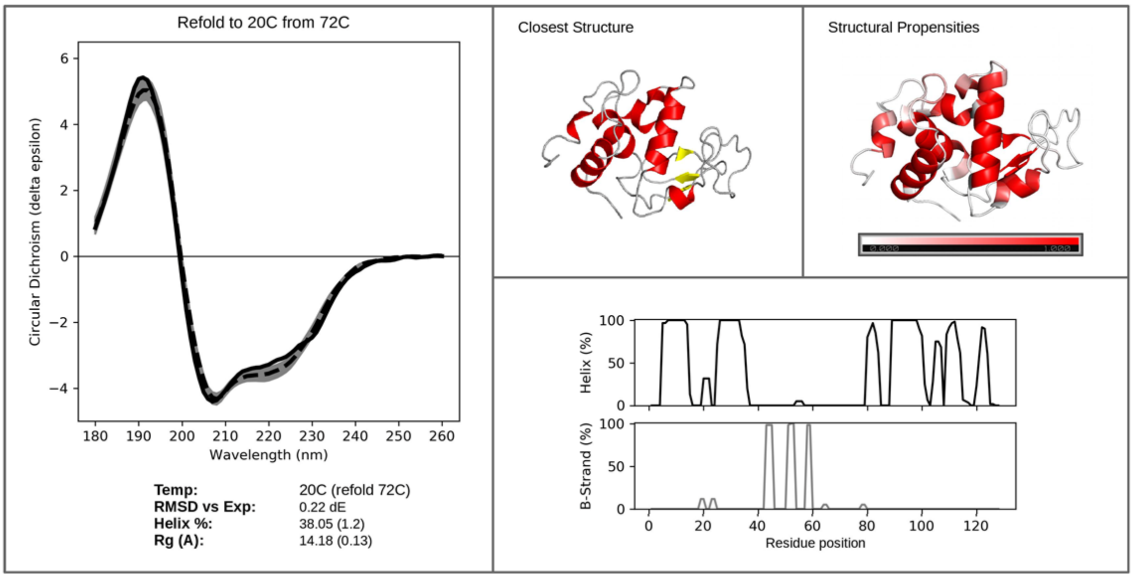

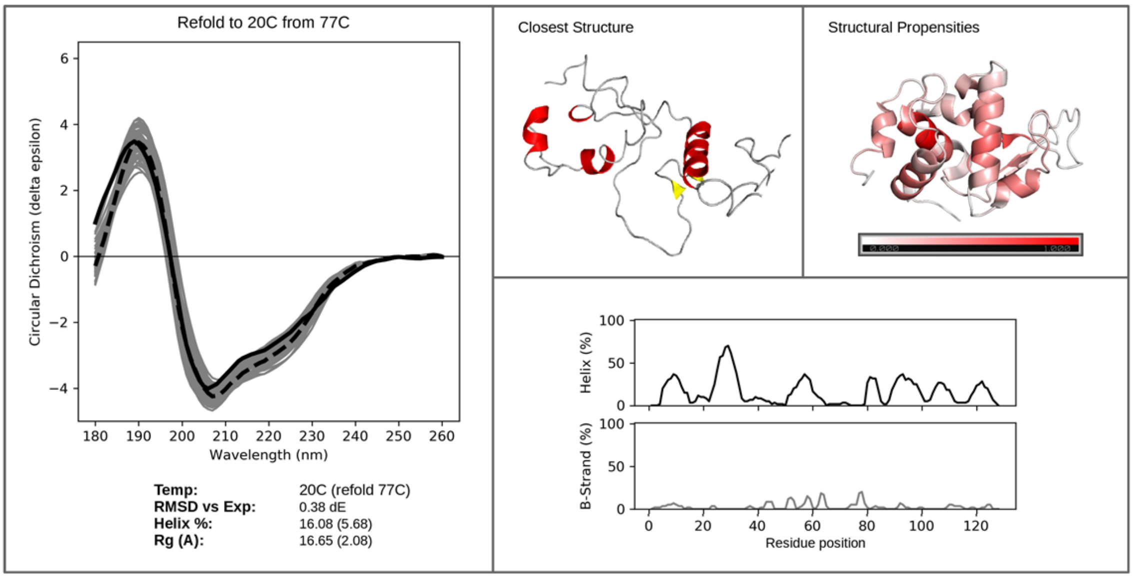

Figure S2: Representative sets produced from fitting of PDBMD2CD predictions of MD-derived structures of lysozyme to experimental CD spectra from 20 ^{O}C to 77 ^{O}C, with refolds to 20 ^{O}C from 72 ^{O}C and 77 ^{O}C. For each temperature, the structures that produced the 60 closest predictions to the experimental spectra (assessed by RMSD) formed a representative set of conformations. A plot of the experimental spectra (black, solid) compared to the 60 predictions (grey) and the average prediction (black, dashed) is shown on the left of each panel. Below the CD plot, the temperature, the RMSD of the average prediction vs the experimental spectra, the average helix percentage of the set and the average radius of gyration (Rg) of the set is detailed. The structure that produced the closest prediction in each set is shown as a "representative structure" for the subset. On the top-right is the native structure of hen egg-white lysozyme from PDB entry 2VB1 coloured according to its per residue, combined helical and beta sheet propensity – this is represented by a gradient from white to red, where white indicates

0% structural propensity for that residue in the set, red 100%. Finally, two plots showing per-residue helix (top) and strand (bottom) propensity can be seen in the bottom right.

PDB ID	PCDDB ID		
1ed9	CD0000002000	1fa2	CD0000009000
1nls	CD0000020000	1m8u	CD0000025000
193l	CD0000045000	3est	CD0000031000
1elp	CD0000024000	1ba7	CD0000065000
2gif	CD0000100000	1dot	CD0000051000
1a49	CD0000061000	1hzx	CD0000123000
2cga	CD0000006000	2nop	CD0000099000
1blf	CD0000042000	1qfe	CD0000028000
1dgf	CD0000017000	1rh5	CD0000124000
3pmg	CD0000057000	1cbj	CD0000068000
3pgk	CD0000058000	7tim	CD0000070000
2dhq	CD0000029000	1ado	CD0000001000
3rn3	CD0000063000	1ppn	CD0000052000
1b8e	CD0000011000	2wjn	CD0000122000
1ubi	CD0000071000	1les	CD0000043000
1nek	CD0000126000	1lin	CD0000013000
2bb2	CD0000022000	1ova	CD0000050000
1xl4	CD0000111000	1ymb	CD0000047000
1t5s	CD0000125000	3dni	CD0000030000
1fcp	CD0000108000	2oar	CD0000115000
5cha	CD0000005000	1une	CD0000059000
1bgl	CD0000010000	1igt	CD0000039000
1l7v	CD0000103000	1hda	CD0000037000
1air	CD0000054000	1fep	CD0000107000
3jqo	CD0000120000	2a65	CD0000113000
1cf3	CD0000033000	2psg	CD0000055000
1a6m	CD0000048000	1hrc	CD0000021000
2j58	CD0000128000	1ax8	CD0000044000
1rhs	CD0000062000	1j95	CD0000110000
1be3	CD0000105000	1nkz	CD0000114000
1hnn	CD0000060000	1a0s	CD0000127000
1bn6	CD0000036000	2vdf	CD0000119000
1ofs	CD0000053000	1hc9	CD0000004000
1ha4	CD0000026000	1kcw	CD0000018000
1hk0	CD0000027000	1k6j	CD0000049000
1thw	CD0000069000	2nr9	CD0000109000
1gpb	CD0000035000	1nqh	CD0000102000
1pcr	CD0000121000	4gcr	CD0000023000

2cts	CD0000019000	1ha7	CD0000012000
1n5u	CD0000038000		
1kpk	CD0000104000		
2cfq	CD0000112000		
2dyr	CD0000106000		
1qhj	CD0000101000		

Table S1: The 83 proteins in the reference set. Shown are the PDB IDs and Protein Circular Dichroism Data Bank (PCDDB) IDs of each reference set protein used to train PDBMD2CD. The spectra for each protein can be found by searching for its ID on the PCDDB website.

**DSSP**	**Least squares**	**Linear combination**
H	H1	H1
G	H2	H2
I	O	I
E	AP1, AP2, P	AP1, AP2, P
T	T	T
S	O	O
B	O	B
No Class/C	O	O

Table S2: Mapping of DSSP classes to the classifications used in the two predictive models used in PDBMD2CD.

PDB ID	PCDDB ID	Protein Name
1q5u	CD0003897000	human dUTPase
2y3z	CD0003898000	3-isopropylmalate dehydrogenase
1qlp	CD0003890000	Alpha-1-antitrypsin
2ccm	CD0004676000	Calexcitin
1sr5	CD0003889000	Antithrombin-III
4kyp	CD0004244000	Bj-xtrIT
1ecz	CD0003896000	Ecotin
2yxf	CD0003894000	Beta-2-microglobulin

Table S3: The eight proteins used as the Test set for this package as chosen from the PCDDB (3)

SUPPLEMENTARY REFERENCES

1. Abdul-Gader,A., Miles,A.J. and Wallace,B.A. (2011) A reference dataset for the analyses of membrane protein secondary structures and transmembrane residues using circular dichroism spectroscopy. *Bioinformatics,* 27, 1630-1636. https://doi.org/10.1093/bioinformatics/btr234

2. Whitmore, L. and Wallace, B.A. (2008) Protein Secondary Structure Analyses from Circular Dichroism Spectroscopy: Methods and Reference Databases. *Biopolymers*, 89, 392-400. (PDF)

3. Whitmore,L., Miles,A.J., Mavridis,L., Janes,R.W. and Wallace,B.A. (2017) PCDDB: new developments at the Protein Circular Dichroism Data Bank. *Nucleic Acids Res*., 45(D1), D303-D307. http://nar.oxfordjournals.org/content/45/D1/D303

4. Woollett,B., Whitmore,L., Janes,R.W. and Wallace,B.A. (2013) ValiDichro: a website for validating and quality control of protein circular dichroism spectra. *Nucleic Acids Res*., 41(W1), W417–W421. https://doi.org/10.1093/nar/gkt287

5. Lees,J.G., Miles,A.J., Wien,F. and Wallace,B.A. (2006) A reference database for circular dichroism spectroscopy covering fold and secondary structure space. *Bioinformatics,* 22, 1955-1962. http://www.ncbi.nlm.nih.gov/pubmed/16787970

6. Kabsch.W. and Sander,C. (1983) Dictionary of protein secondary structure: pattern recognition of hydrogen-bonded and geometrical features. *Biopolymers*, 22, 2577-637. doi:10.1002/bip.360221211. PMID 6667333.

7. Manavalan,P. and Johnson,W. (1983) Sensitivity of circular dichroism to protein tertiary structure class. *Nature*, 305**,** 831–832. https://doi.org/10.1038/305831a0

8. Wu,J., Yang,J.T. and Wu,C.S.C. (1992) β-II conformation of all-β proteins can be distinguished from unordered form by circular dichroism. *Analytical Biochem*., 200, 359-364. https://doi.org/10.1016/0003-2697(92)90479-Q.

9. Micsonai,A., Wien,F., Bulyáki,E., Kun,J., Moussong,E., Lee,Y.H., Goto,Y., Réfrégiers,M. and Kardos,J. (2015) Accurate secondary structure prediction and fold recognition for circular dichroism spectroscopy, *Proc. Nat. Acad. Sci.*, 112, E3095-E3103. doi/10.1073/pnas.1500851112

10. Virtanen,P., Gommers,R., Oliphant,T.E., Haberland,M., Reddy,T., Cournapeau,D., Burovski,E., Peterson,P., Weckesser,W., Bright,J., van der Walt,S.J., Brett,M., Wilson,J., Millman,K.J., Mayorov,N., Nelson,A.R.J., Jones,E., Kern,R., Larson,E., Carey,C.J., Polat,İ., Feng,Y., Moore,E.W., VanderPlas,J., Laxalde,D., Perktold,J., Cimrman,R., Henriksen,I., Quintero,E.A., Harris,C.R., Archibald,A.M.,

Ribeiro,A.H., Pedregosa,F., van Mulbregt,P. and SciPy 1.0 Contributors. (2020) SciPy 1.0: Fundamental Algorithms for Scientific Computing in Python. *Nature Methods*, 17, 261-272. doi.org/10.1038/s41592-019-0686-2

11. Jo,S., Kim,T., Iyer,V.G. and Im,W. (2008) CHARMM-GUI: A Web-based Graphical User Interface for CHARMM. *J. Comput. Chem*., 29, 1859-1865. doi.org/10.1002/jcc.20945

12. Lee,J., Cheng,X., Swails,J.M., Yeom,M.S., Eastman,P.K. Lemkul,J.A., Wei,S., Buckner,J., Jeong,J.C., Qi,Y., Jo,S., Pande,V.S., Case,D.A., Brooks III,C.L., MacKerell Jr,A.D., Klauda,J.B. and Im,W. (2016) CHARMM-GUI Input Generator for NAMD, GROMACS, AMBER, OpenMM, and CHARMM/OpenMM Simulations using the CHARMM36 Additive Force Field. *J. Chem. Theory Comput*., 12, 405-413. doi.org/10.1021/acs.jctc.5b00935

13. Abraham,M.J., Murtola,T., Schulz,R., Páll,S., Smith,J.C., Hess,B. and Lindahl,E. (2015) GROMACS: High performance molecular simulations through multi-level parallelism from laptops to supercomputers, *SoftwareX*, 1–2, 19-25. https://doi.org/10.1016/j.softx.2015.06.001.

14. Meersman,F., Atilgan,C., Miles,A.J., Bader,R., Shang,W.F., Matagne,A., Wallace,B.A. and Koch,M.H.J. (2010) Consistent Picture of the Reversible Thermal Unfolding of Hen Egg-White Lysozyme from Experiment and Molecular Dynamics. *Biophysical J.,* 99, 2255-2263. https://doi.org/10.1016/j.bpj.2010.07.060
